# Cocaine Modulates Locomotion Behavior in *C. elegans*

**DOI:** 10.1371/journal.pone.0005946

**Published:** 2009-06-17

**Authors:** Alex Ward, Vyvyca J. Walker, Zhaoyang Feng, X. Z. Shawn Xu

**Affiliations:** 1 Life Sciences Institute, University of Michigan, Ann Arbor, Michigan, United States of America; 2 Department of Molecular and Integrative Physiology, University of Michigan, Ann Arbor, Michigan, United States of America; 3 Neuroscience Graduate Program, University of Michigan, Ann Arbor, Michigan, United States of America; 4 Program in Biomedical Sciences, University of Michigan, Ann Arbor, Michigan, United States of America; Massachusetts General Hospital/Harvard Medical School, United States of America

## Abstract

Cocaine, a potent addictive substance, is an inhibitor of monoamine transporters, including DAT (dopamine transporter), SERT (serotonin transporter) and NET (norepinephrine transporter). Cocaine administration induces complex behavioral alterations in mammals, but the underlying mechanisms are not well understood. Here, we tested the effect of cocaine on *C. elegans* behavior. We show for the first time that acute cocaine treatment evokes changes in *C. elegans* locomotor activity. Interestingly, the neurotransmitter serotonin, rather than dopamine, is required for cocaine response in *C. elegans*. The *C. elegans* SERT MOD-5 is essential for the effect of cocaine, consistent with the role of cocaine in targeting monoamine transporters. We further show that the behavioral response to cocaine is primarily mediated by the ionotropic serotonin receptor MOD-1. Thus, cocaine modulates locomotion behavior in *C. elegans* primarily by impinging on its serotoninergic system.

## Introduction

Cocaine is a plant alkaloid derived from coca plant leaves and represents a major drug of abuse. In animal models, acute administration of cocaine evokes changes in locomotor activity, grooming, and feeding, and can induce uncontrolled repetitive behaviors [1]. At the cellular level, cocaine elevates extracellular monoamine levels by inhibiting monoamine reuptake transporters, including DAT, SERT and NET [2], [3]. By acting on their cognate receptors, monoamines elicit both short-term and long-lasting alterations in the nervous system, which ultimately lead to the development of drug dependence [4].

While dopamine is generally believed to be a principal neurotransmitter functioning in the mesolimbic dopamine system to mediate drug dependence, ample evidence suggests that other neurotransmitter systems are also required for the expression of drug addiction behaviors [5], [6], [7], [8], [9]. In particular, serotonin is believed to play an important role in mediating the reinforcing effects of cocaine [8], [9]. For example, the induction of conditioned place preference (CPP) by cocaine is normal in DAT knockout mice, but is eliminated in mice lacking both DAT and SERT [10], [11]. Further, it has been shown that that DAT knockout mice can still self-administer cocaine [12], [13], though a recent study has challenged this finding [14]. Therefore, to better understand the mechanistic underpinnings of drug addiction, and to develop new therapeutic interventions, a greater knowledge of the genes and molecules regulating cocaine's behavioral effects is required.

Despite their simplicity, invertebrate model organisms such as *C. elegans* and *Drosophila* are widely used in neurobiology and have yielded novel insights into relatively complex behavioral phenomena, including drug dependence [15]. Indeed, recent work in *C. elegans* has identified new genes involved in alcohol intoxication and nicotine dependence, while *Drosophila* has proved to be a powerful model system for the study of alcohol tolerance/intoxication and cocaine sensitivity [16], [17], [18], [19], [20]. Importantly, *C. elegans* and *Drosophila* share with mammals many of the same neurotransmitters, synaptic machinery, transporters, ion channels, and signal transduction mechanisms [21]. Furthermore, the major genes found to be involved in drug dependence are conserved in these organisms [19], [21]. The powerful genetics of invertebrate models, combined with their short generation time, make these organisms a valuable resource for the study of basic mechanisms underlying drug-induced behaviors.

In the present study, we tested the effect of cocaine on *C. elegans* locomotion behavior. We find that acute cocaine treatment alters its locomotor activity. This behavioral response to cocaine is mediated by serotonin. We also provide genetic evidence that the molecular target of cocaine is the *C. elegans* SERT MOD-5. We further show that the response to cocaine in *C. elegans* requires the ionotropic serotonin receptor MOD-1.

## Results

### Acute cocaine exposure induces a hypolocomotor response in *C. elegans*

**Figure 1:**
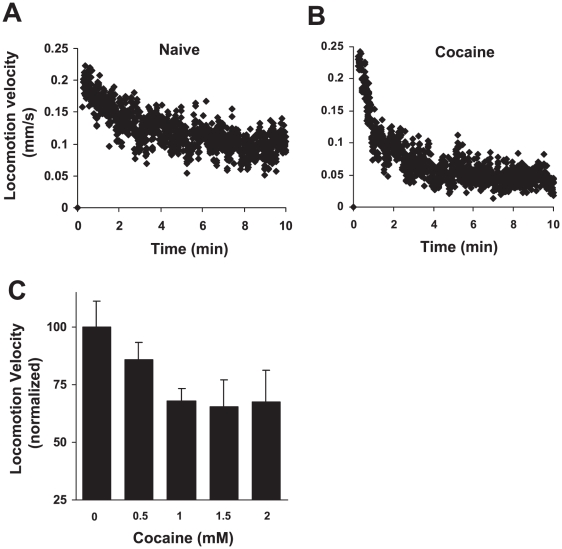
Cocaine induces a hypolocomotor response in *C. elegans*. (A–B) Worms were tracked on plates without cocaine (A) or with 1.5 mM cocaine. Shown are traces averaged from 10 worms. (C) Worms respond to cocaine in a dose-dependent manner. 10 worms were assayed for each concentration. Error bars: SEM.

In rodent and fly models, acute administration of cocaine to naïve animals alters locomotor activity, a behavioral parameter commonly used for the study of cocaine responses in mammals [22], [23]. To determine whether cocaine modulates motor behavior in the genetic model *C. elegans*, we employed an automated worm tracking system that records worm locomotion and reports its activity in real time [24]. Previous work in our lab using this tracking system has demonstrated that naïve worms transferred to a new plate display a gradual decline in locomotion velocity until reaching a relatively steady state or basal speed [19]. This finding is attributed to the worm's locomotor response to a new environment (i.e. a new plate with fresh bacteria), and is consistent with previous work [25]. In this study, we assayed locomotion behavior after transferring worms to plates containing cocaine in the agar. The response to cocaine was quantified as average locomotion velocity during the tracking period. Using this measure, we found that cocaine significantly decreased average locomotion velocity in wild-type worms in a dose-dependent manner, indicating that cocaine can evoke a hypolocomotor response in *C. elegans* (Figure 1 and Supplementary Table S1).

### The acute response to cocaine requires serotonin

**Figure 2:**
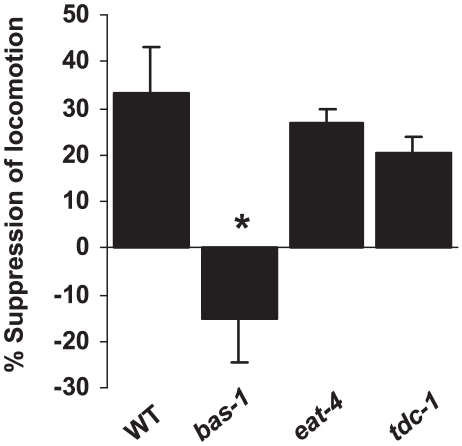
The cocaine-induced hypolocomotor response requires serotonin and/or dopamine. Data are quantified as percentage suppression of locomotion velocity between naïve and cocaine-treated conditions for each genotype. *p<0.01 (ANOVA with the Bonferroni test comparing mutants and WT). Error bars: SEM.

Having developed a worm model for acute cocaine response, we next set out to determine which genes mediate the locomotor response to cocaine in *C. elegans*. In mammals, cocaine acts on the nervous system by inhibiting monoamine reuptake transporters [2], [3]. *C. elegans* possesses serotoninergic, dopaminergic and tyraminergic neurons, but lacks norepinephrine, and instead has octopamine [15], [26]. To identify which neurotransmitter(s) mediates the hypoactive effects of cocaine in *C. elegans*, we tested neurotransmitter-defective mutants for their response to cocaine. These include *bas-1* (which encodes an aromatic amino acid decarboxylase (AADC) required for the production of serotonin and dopamine) [27], *tdc-1* (which encodes an aromatic-L-amino-acid/L-histidine decarboxylase required for making tyramine and octopamine) [26], and *eat-4* (which encodes a vesicular glutamate transporter important for glutamate-mediated neurotransmission) [28]. Acetylcholine and GABA deficient mutants were not tested because mutants deficient in these two neurotransmitters are severely uncoordinated, and thus not suitable for locomotion assays [29], [30]. While *tdc-1* and *eat-4* mutants behaved similarly to wild-type worms in response to cocaine treatment, *bas-1* animals completely suppressed the hypoactive response to cocaine (Figure 2). This finding indicates that serotonin and/or dopamine regulates the hypoactive effects of cocaine in worms.

We noticed that besides lacking the cocaine-induced hypolocomotor response, *bas-1* worms were slightly hyperactive in response to cocaine (Figure 2). This suggests the presence of an additional target for cocaine that is independent of serotonin and dopamine. Given that this hyperlocomotor response is independent of these two neurotransmitters and also not manifested in wild-type worms, we decided to focus on the hypolocomotor response induced by cocaine.

To distinguish whether serotonin or dopamine has a role in regulating the hypolocomotor response to cocaine, we tested several serotonin- and dopamine-specific mutants, including *tph-1* and *cat-2. tph-1* encodes the sole *C. elegans* tryptophan hydroxylase required for serotonin synthesis [31], while *cat-2* represents the sole *C. elegans* tyrosine hydroxylase essential for the production of dopamine [32]. We also tested mutants for the presumptive targets of cocaine, namely *mod-5* which encodes the sole *C. elegans* serotonin transporter [33], and *dat-1* which is the sole *C. elegans* dopamine transporter [34]. It has been previously demonstrated that MOD-5 can function as a serotonin transporter in heterolgous systems, and that cocaine can inhibit the activity of MOD-5 in uptaking serotonin [33]. Using a similar approach, DAT-1 has been shown as a dopamine transporter sensitive to cocaine [34].

**Figure 3:**
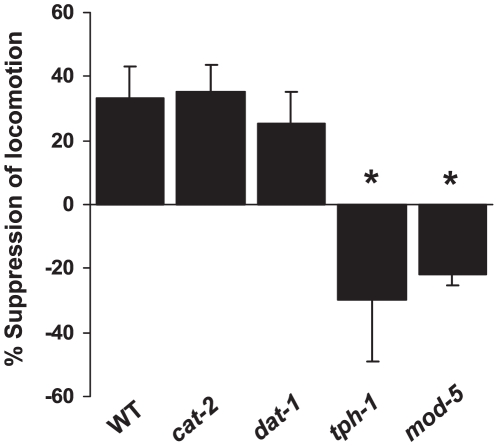
The cocaine-induced hypolocomotor response requires serotonin and depends on SERT/MOD-5. Data are quantified as percentage suppression of locomotion velocity between naïve and cocaine-treated conditions for each genotype. *p<0.002 (ANOVA with the Bonferroni test comparing mutants and WT). Error bars: SEM.

Surprisingly, *cat-2* mutant worms, which are deficient in dopamine synthesis, responded like wild-type to acute cocaine treatment (Figure 3). Worms lacking the dopamine transporter DAT-1 also responded normally to cocaine (Figure 3). Thus, it appears that dopamine is not required for the hypolocomotor response to cocaine in *C. elegans*.

By contrast, *tph-1* mutant worms, which are devoid of serotonin synthesis, lacked the hypolocomotor response to cocaine (Figure 3), indicating that serotonin is required for this cocaine response. Furthermore, mutant worms lacking MOD-5, the *C. elegans* SERT, failed to exhibit the hypolocomotor response to cocaine (Figure 3), demonstrating a critical role for SERT in cocaine sensitivity in *C. elegans*. Taken together, these observations suggest that serotonin mediates the hypolocomotor response to cocaine in worms, and support the notion that cocaine targets monoamine transporters. Our data are also consistent with previous findings that exogenous serotonin inhibits locomotor activity in *C. elegans* [35], [36], [37].

### The ionotropic serotonin receptor MOD-1 is required for cocaine response

**Figure 4:**
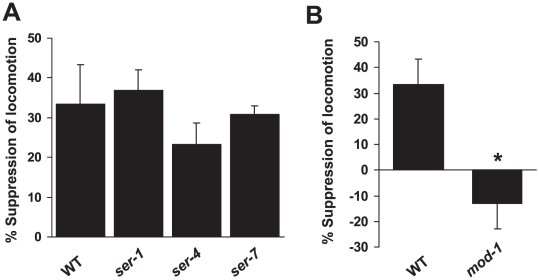
The cocaine-induced hypolocomotor response requires the ionotrpic serotonin receptor MOD-1. (A) The metabotropic serotonin receptor mutants *ser-1, ser-4* and *ser-7* do not show a severe defect in the cocaine-induced hypolocomotor response. (B) The ionotropic serotonin receptor mutant *mod-1* lacks the hypolocomotor response induced by cocaine. *p = 0.01 (ANOVA). Error bars: SEM.

Serotonin exerts its effects by acting on serotonin receptors. Thus, to further understand how serotonin mediates cocaine response in *C. elegans*, we examined serotonin receptor mutants for their response to cocaine. First, we tested the seven-transmembrane metabotropic serotonin receptor mutants *ser-1*, *ser-4* and *ser-7* [38], [39], [40], [41], [42], [43], [44] (Figure 4). Interestingly, none of the tested metabotropic serotonin receptor mutants displayed a severe defect in response to cocaine (Figure 4A). Nevertheless, we note that there could be functional redundancy between these serotonin receptors and other *ser* family genes in the *C. elegans* genome might encode additional serotonin receptors. Thus, it remains possible that metabotropic serotonin receptors may play a role in mediating cocaine response.

In addition to metabotropic serotonin receptors, the *C. elegans* genome encodes an ionotropic serotonin receptor, MOD-1 [37]. MOD-1 can function as a serotonin-gated Cl^−^ channel in heterologous systems and is important for serotonin-mediated olfactory learning and sensation [43], [45]. MOD-1 also plays a critical role in serotonin-dependent enhanced slowing response in which it functions as a downstream target of MOD-5/SERT [33].

We thus tested *mod-1* mutant worms. We found that the hypolocomotor response to cocaine was absent in *mod-1* mutant animals (Figure 4B), indicating that *mod-1* is required for cocaine response in *C. elegans*. This data is also consistent with the finding that *mod-1* mutant worms are resistant to exogenous serotonin-induced paralysis [37].

## Discussion

In this study, we have shown that *C. elegans* responds to acute cocaine treatment by reducing locomotor activity, a behavioral response that is mediated by the neurotransmitter serotonin. This observation is consistent with previous studies showing that acute administration of serotonin inhibits worm locomotion [35], [36], [37]. In addition, we provide genetic evidence that the molecular target of cocaine is the *C. elegans* SERT *mod-5*. These results also suggest that the observed cocaine response is not due to a non-specific local anesthetic effect of cocaine, which primarily results from its blockade of voltage-gated sodium channels.

Locomotion is probably not the only worm behavior that can be modulated by cocaine. In *C. elegans*, serotonin regulates a wide variety of behaviors, including egg-laying, feeding, chemosensation, male turning, and learning and memory [27], [43], [44], [46]. Thus, it remains possible that cocaine may also modulate other types of worm behaviors.

In rodents, cocaine can target all major types of monoamine transporters, including DAT, SERT and NET [2], [3]. Surprisingly, we did not detect a significant role for dopamine in cocaine-induced locomotor response, considering that acute dopamine treatment has also been demonstrated to inhibit worm locomotion [47], [48]. Nevertheless, it remains possible that dopamine may play a role in mediating cocaine response in *C. elegans* but such a role is not manifested in our assay.

The response to cocaine in *C. elegans* requires the ionotropic serotonin receptor MOD-1, suggesting MOD-1 as a downstream effector of cocaine. Since MOD-1 is an inhibitory Cl^−^ channel [37], this suggests that the cocaine-induced hypolocomotor response may result from MOD-1-mediated inhibition of locomotion. Indeed, MOD-1 has been shown to mediate serotonin-induced paralysis of *C. elegans* [37]. In rodents, one of the major downstream targets of cocaine is the 5-HT_1A_-receptor, which couples via Gi/Go to a hyperpolarizing K^+^ conductance, and is thus inhibitory [8]. Therefore, in both worms and mammals cocaine appears to evoke a serotonin-mediated response through inhibition of neurotransmission.

Our findings shed light on questions surrounding the involvement of serotonin in mediating the behavioral effects of psychostimulant drugs such as cocaine [10], [12], [14]. A growing body of evidence demonstrates that in addition to dopamine, serotonin plays an important role in mediating behavioral and addictive effects of cocaine [8], [9]. Our results from *C. elegans* also support a critical role for serotonin in cocaine responses. Although at the behavioral level cocaine elicits distinct responses in worms and mammals (hypo- vs. hyper-locomotor response), at the molecular level this drug impinges on similar types of genes and pathways in both organisms, suggesting that *C. elegans* may be used to study the mechanisms by which serotoninergic signaling regulates cocaine responses.

## Materials and Methods

### Strains

The following mutant alleles were used in the study: MT7988: *bas-1(ad446)*; GR1321: *tph-1(mg280)*; CB1112: *cat-2(e1112)*; MT13113: *tdc-1(n3419)*; DA572: *eat-4(ad572)*; TQ328: *dat-1(tm903)*; MT9772: *mod-5(n3314)*; MT9668: *mod-1(ok103)*; DA1814: *ser-1(ok345)*; AQ866: *ser-4(ok512)*; DA2100: *ser-7(tm1325)*.

### Behavioral and statistical analysis

Locomotion behaviour was analyzed using an automated worm tracking system as previously described [19], [24], [49], [50]. In brief, L4 hermaphrodites were picked 16 hours prior to behavioural analysis. Tracking was performed on NGM plates (lid off) covered with a thin layer of OP50 bacteria spread at 5 min before tracking. Worms analyzed on bacteria-free plates did not exhibit robust cocaine response. The room temperature was maintained at 20–21°C with a relative humidity of 30–40%. Cocaine (1.5 mM) was directly spread on the surface of NGM plates and allowed to diffuse for at least 16 hours. The automated tracking system comprises a stereomicroscope (Zeiss Stemi 2000C), a digital camera (Cohu 7800) to acquire worm images, a digital motion system (Parker Automation) to follow worm movement, and a home-developed software package to control the hardware. Images were grabbed at 2 Hz, and the locomotion velocity at each time point was computed as centroid displacement (mm) per sec and plotted in real time during tracking. The velocity data were also saved as text files and used to calculate the average locomotion velocity during the tracking period (10 min).

Statistical analyses were carried out using KaleidaGraph (Synergy Software, Inc). *P* values were generated by ANOVA with the Bonferroni test. Error bars represent SEM. *P*<0.05 was considered significant.

## Supporting Information

Table S1 Average locomotion velocity in naïve and cocaine-treated worms
(0.39 MB EPS)
